# Two-Stage Liver and Tumor Segmentation Algorithm Based on Convolutional Neural Network

**DOI:** 10.3390/diagnostics11101806

**Published:** 2021-09-29

**Authors:** Lu Meng, Qianqian Zhang, Sihang Bu

**Affiliations:** College of Information Science and Engineering, Northeastern University, Shenyang 110000, China; 2070865@stu.neu.edu.cn (Q.Z.); 1970708@stu.neu.edu.cn (S.B.)

**Keywords:** medical image segmentation, deep learning, convolutional neural network, liver tumor, attention mechanism

## Abstract

The liver is an essential metabolic organ of the human body, and malignant liver tumors seriously affect and threaten human life. The segmentation algorithm for liver and liver tumors is one of the essential branches of computer-aided diagnosis. This paper proposed a two-stage liver and tumor segmentation algorithm based on the convolutional neural network (CNN). In the present study, we used two stages to segment the liver and tumors: liver localization and tumor segmentation. In the liver localization stage, the network segments the liver region, adopts the encoding–decoding structure and long-distance feature fusion operation, and utilizes the shallow features’ spatial information to improve liver identification. In the tumor segmentation stage, based on the liver segmentation results of the first two steps, a CNN model was designed to accurately identify the liver tumors by using the 2D image features and 3D spatial features of the CT image slices. At the same time, we use the attention mechanism to improve the segmentation performance of small liver tumors. The proposed algorithm was tested on the public data set Liver Tumor Segmentation Challenge (LiTS). The Dice coefficient of liver segmentation was 0.967, and the Dice coefficient of tumor segmentation was 0.725. The proposed algorithm can accurately segment the liver and liver tumors in CT images. Compared with other state-of-the-art algorithms, the segmentation results of the proposed algorithm rank the highest in the Dice coefficient.

## 1. Introduction

With the development of computer technology, computer-aided technology has been a popular method to analyze medical images, which can assist clinicians in detecting and segmenting tumor lesion regions from normal tissues. Computer-aided diagnosis eliminates human subjective influence and avoids unnecessary diagnosis errors, improving the accuracy of lesion region identification and improving doctors’ work efficiency.

The liver is an essential metabolic organ of the human body, in charge of metabolism, digestion, and detoxification. However, malignant liver tumors seriously affect and threaten human lives. In 2018, global cancer statistics reported approximately 840,000 liver cases and 780,000 related deaths [1]. Traditionally, radiologists have to watch the liver CT slices one by one to find the liver tumors, which is not only very time-consuming and laborious but also easy to make errors due to fatigue or subjective judgments. Therefore, there is an urgent need for automatic liver tumor detection and segmentation algorithms to assist clinicians.

Since 2014, deep learning has performed well in image detection and segmentation [2]. Compared with traditional methods, the convolutional neural network (CNN) has been proven effective in processing images. Especially the fully convolutional neural network (FCN) has achieved excellent results in medical image identification, classification, and segmentation [3]. Many researchers have used FCN-related algorithms to segment liver and tumors, among which the best model is U-Net [4], which consists of a contracting path and an expansive path, which makes it have the U-shaped architecture.

There are two main branches for medical image segmentation based on FCN, which are 2D-FCN and 3D-FCN, and the main difference between the two is the dimension of the convolution kernel and feature maps. Ben-Cohen et al. [5] used a fully convolutional structure for liver segmentation and liver metastases detection in CT images. They discarded the classifier layer and replaced the fully connected layer with a convolutional layer to detect tumors on the 2D CT image. Sun et al. [6] proposed a new automatic liver tumor segmentation method named multi-channel fully convolutional network (MC-FCN). Compared with single-channel FCN, MC-FCN has three FCN channels, with independent training parameters used for image feature extraction and parameter training. Chlebus et al. [7] segmented liver tumors based on 2D fully convolutional neural networks, which transferred the feature map via long-distance skip connections to restore the detailed information lost in the spatial downsampling.

Some researchers use a 3D convolution kernel to replace the 2D convolution kernel to obtain the three-dimensional features maps of medical images. Lu et al. [8] combined 3D CNN with the image segmentation algorithm to effectively detect the liver regions. They evaluated the algorithm on the two public data sets of MICCAI-Sliver07 and 3Dircabd. Compared with the existing automatic liver segmentation algorithm, this method has higher segmentation accuracy and improves doctors’ work efficiency without user interaction. Aqyyum et al. [9] proposed a 3D hybrid model for CT images, which consisted of a three-dimensional residual network, spatial squeeze module, and excitation module. This algorithm performed well for the segmentation of liver and large tumor regions, but the detection of small tumor regions was not accurate. Jiang et al. [10] proposed a 3D convolutional neural network structure composed of multiple attention hybrid connection modules and soft attention modules. The network focused on learning the features of the tumor and background. The algorithm was tested on the 3DIRCADb data set, and the tumor segmentation accuracy of this algorithm was 0.62. Especially for small tumor segmentation.

Although the existing algorithms performed well in segmenting liver and liver tumors, there are still some shortcomings: (1) they focus on either 2D features or 3D features of the liver CT images, and ignore the hybrid features from 2D and 3D; and (2) segmentation performance of small liver tumors is poor, which is caused by the small proportion of small liver tumor in the CT image and low gradient between the liver tumor and background.

To address these shortcomings, we presented several solutions: (1) we designed a two-stage densely connected UNet (DCUNet) for liver and liver tumor segmentation, which consists of two stages, and we focused on both 2D and 3D features in the proposed algorithm; and (2) we added an attention mechanism to the neural network architecture to learn the multi-scale features of small tumors in the liver.

## 2. Method

### 2.1. Overall Process

**Figure 1:**
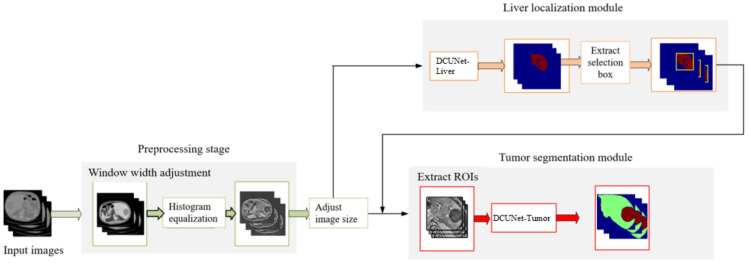
The overall flowchart of the algorithm.

The overall flow chart of the proposed algorithm is shown in Figure 1, which is composed of four main steps:

(1) In the preprocessing stage, the original CT image window width is adjusted to enhance the contrast of the liver region. We use the histogram equalization to extend the processed CT image pixels nonlinearly. The operation makes the pixels evenly distributed and highlights the features of the tumor region.
(2) In the first stage, DCUNet-Liver is used to obtain the segmentation results of the liver region.
(3) In the third stage, according to the liver segmentation results, the detailed 2D features in the CT images are extracted and fused with the 3D spatial features to optimize the segmentation results of liver tumors.

### 2.2. Stage One: DCUNet-Liver for Liver Segmentation

**Figure 2:**
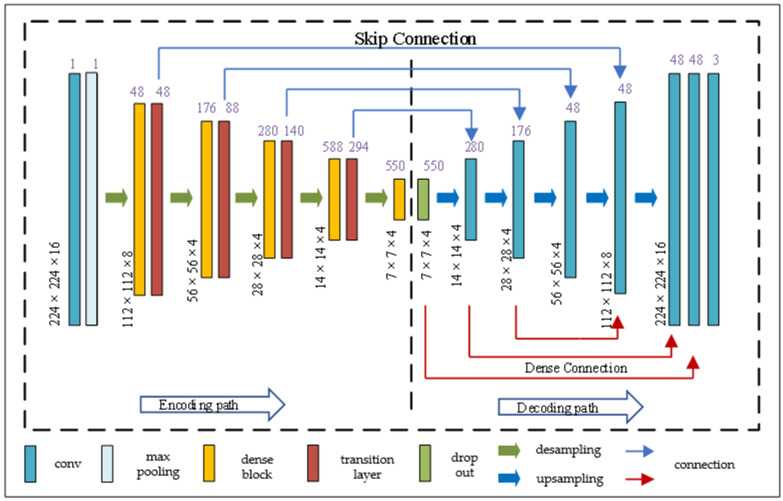
DCUNet-Liver network structure diagram.

As shown in Figure 2, the structure of DCUNet-Liver consists of two parts. The left part of the dotted line is the encoding part, which is mainly composed of dense blocks and transition layers; the right part of the dotted line is the decoding part. In the network structure of DCUNet-Liver, the convolution layer, max-pooling layer, drop out, and upsampling are all regular operations in deep learning methods, and the details of the dense block and transition layer are elaborated below.

**Figure 3:**
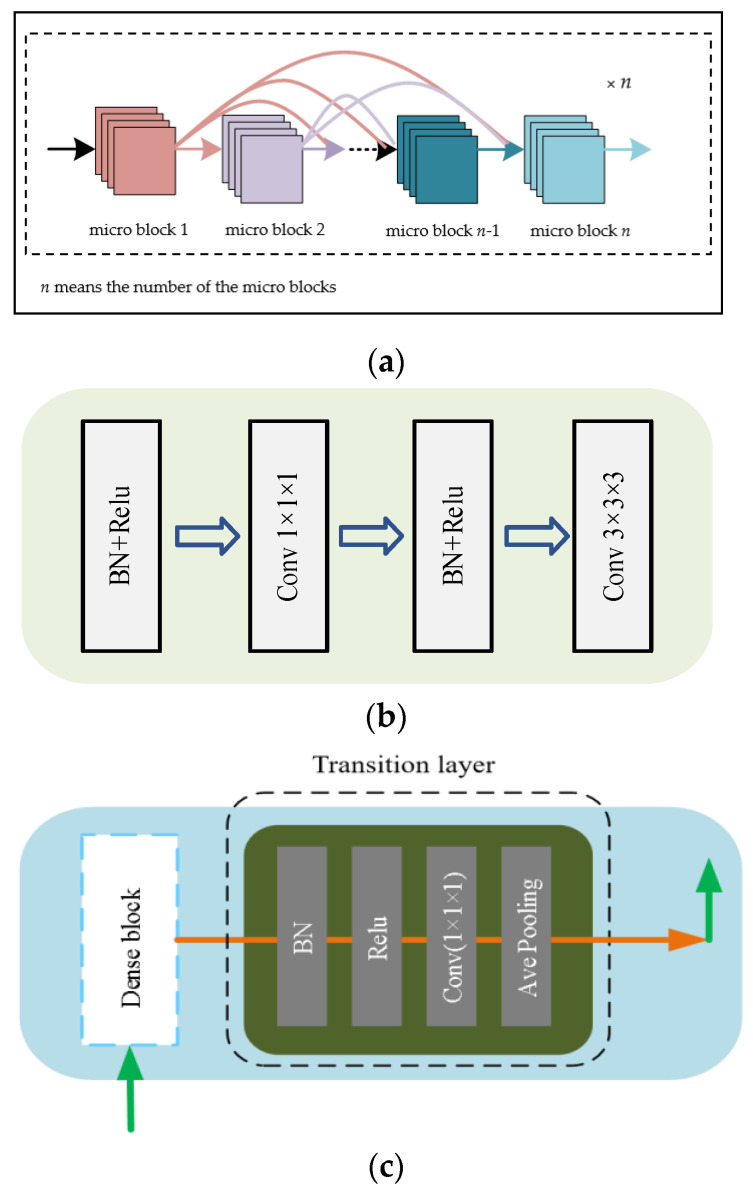
The structure of the dense block [11], micro block, and transition layer: (**a**) the dense block network structure diagram; (**b**) the micro block network structure diagram; (**c**) the transition layer structure diagram.

There are four dense blocks in the DCUNet-Liver, and in each dense block, the number of micro blocks is 4, 6, 14, and 8, respectively. As shown in Figure 3a, it is the structure of the dense block. Each dense block contains multiple micro blocks. The output of each micro block is connected to all subsequent micro blocks by residual connections. For example, the output of micro block #1 is connected to micro block #2, micro block #3, until micro block #*n*. The main purpose of the micro block is transferring the feature maps of one block to all others, and can increase the nonlinearity of the whole network and accelerate the training process of the network.

As shown in Figure 3b, it is the structure of the micro block. In the dense block, each micro block generates *K* feature maps, and we set the parameter *K* as the growth rate and use it to control the number of feature maps generated by the dense block. For example, in the first stage, *K* is 32, which means that if the input of a micro block is *M* feature maps, then the output of the micro block is *M* + 32 feature maps. 

Therefore, when the neural network contains multiple dense blocks, the number of feature maps increases, which can significantly increase the number of parameters, and make it harder to train the network. To solve the problem, the proposed algorithm adds a transition layer at the output of each dense block, and the network structure of the transition layer is shown in Figure 3c.

**Figure 4:**
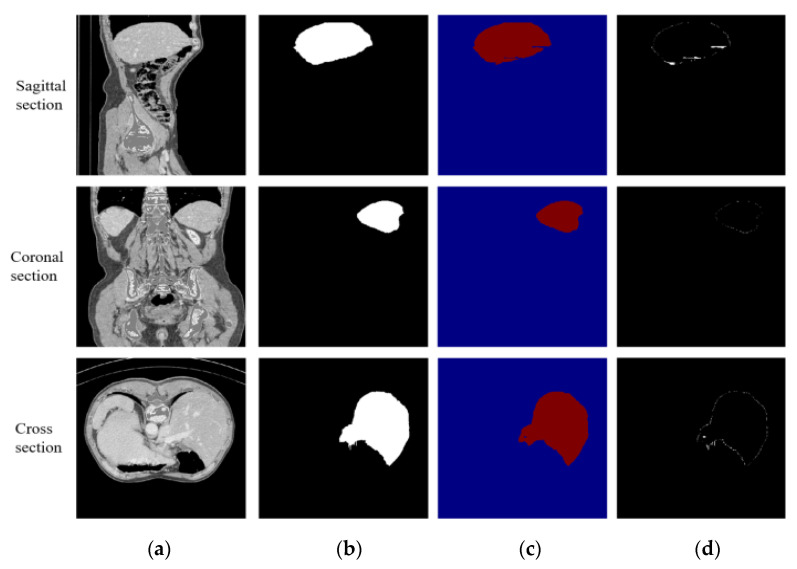
The segmentation results of liver localization module: (**a**) the original CT images; (**b**) the real segmentation results; (**c**) the segmentation results of DCUNet-Liver; (**d**) the difference images.

In the first stage, DCUNet-Liver contains four dense blocks; therefore, four transition layers are added, and each transition layer is composed of batch normalization (BN), an activation function, convolutional layer (1 × 1 × 1), and pooling layer. The role of the transition layer is to reduce the redundant feature maps generated by dense blocks and downsample the feature maps. The segmentation results obtained by the liver localization module on the sagittal, coronal, and cross-sections are shown in Figure 4.

### 2.3. Stage Two: DCUNet-Tumor for Liver Tumor Segmentation

**Figure 5:**
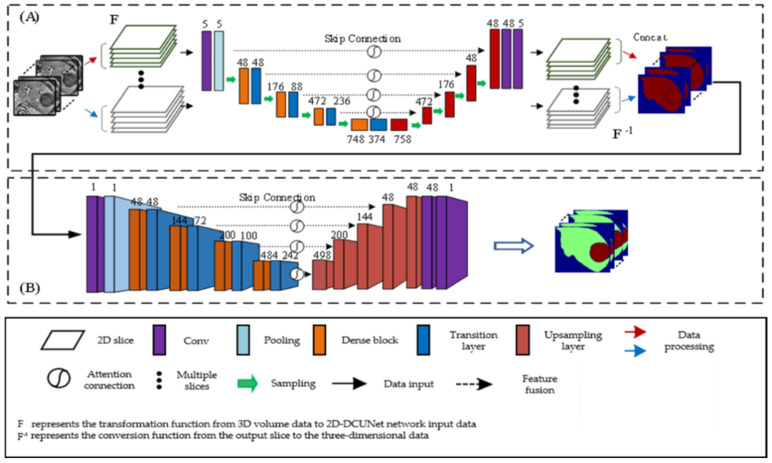
DCUNet-Tumor network structure diagram: (**A**) a two-dimensional U-Net network; (**B**) a three-dimensional U-Net network.

At this stage, based on the accurate segmentation results of the liver region, the tumors in the liver are further detected and segmented. The proposed algorithm adopts the combination of a two-dimensional network and three-dimensional network to fuse the two-dimensional plane features and three-dimensional spatial features of the tumor, so as to realize the accurate segmentation of liver tumor. The structure of DCUNet-Tumor is shown in Figure 5. Taking the segmentation results of the liver region as the input of this stage, firstly, it is processed through a two-dimensional U-Net network, and then the obtained feature maps are sent to the three-dimensional U-Net network for further processing. The 2D plane features of the CT image are combined with 3D spatial information to detect the liver tumor region.

**Figure 6:**
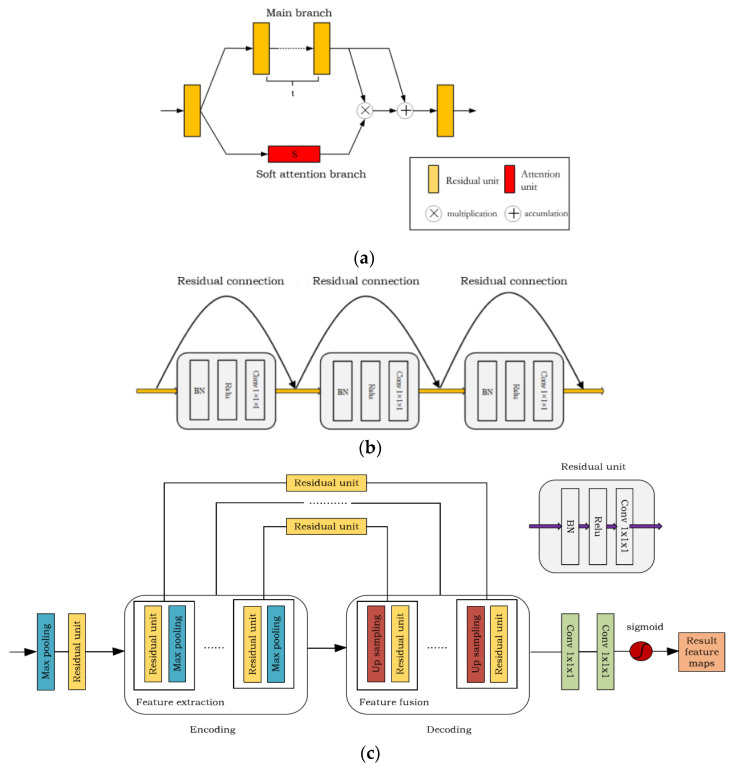
Attention module structure and its main branch and the soft attention branch structure diagram: (**a**) the attention module structure diagram, where t represents the number of residual units in the main branch; (**b**) the main branch structure diagram; (**c**) the soft attention branch structure diagram.

The lesion areas of the liver tumors in different patients vary greatly, and the size, location, and shape of the tumors are different, especially for small tumors, which increases the difficulty of recognition. The convolution neural network will lose the information of small tumors and reduce the segmentation accuracy of tumors when extracting features. To solve this problem, the proposed algorithm adds the attention module to the skip connections of the DCUNet-Tumor network, which makes the neural network pay more attention to the liver tumor areas, so as to improve the segmentation accuracy of liver tumors. The attention module structure is shown in Figure 6a, which includes the main branch and soft attention branch. The main branch structure is a general residual network composed of multiple residual units, which include the BN layer, ReLU activation function, and 1 × 1 convolutional layer, shown in Figure 6b. The soft attention branch is composed of an encoding and decoding architecture, which focuses on extracting the context information of the small tumor areas in the image, shown in Figure 6c.

The purpose of the main branch is to extract the global feature information in the image, such as the background information and liver information. The residual units in the main branch directly propagate features from the previous convolution layers to the rear convolution layer, which solves the problem of gradient disappearance and improves the segmentation performance of the neural network. However, simply accumulating residual units may reduce the network’s performance [12], and the output of the traditional attention module is in Equation (1),

(1) $$H_{i,c}\left(x\right)=M_{i,c}\left(x\right)\times T_{i,c}\left(x\right)$$

where *T*(*x*) represents the feature maps from the main branch, *M*(*x*) represents the feature mask from the soft attention branch, and × represents the element-wise product operation. In our attention module, the feature mask can be used as a feature selector in the forward learning process and as a gradient update filter in the backpropagation process. In the soft attention branch, the mask gradient of the input feature is in Equation (2),

(2) $$\frac{\partial M\left(x,\theta \right)T\left(x,\emptyset \right)}{\partial \emptyset }=M\left(x,\theta \right)\frac{\partial T\left(x,\emptyset \right)}{\partial \emptyset }$$

where θ is the soft attention branch parameter and ∅ is the main branch parameter. However, the range of *M*(*x*) is [0, 1], and if multiple modules are multiplied directly, the value of the feature map will become smaller and smaller, which may hinder the performance of the neural network. To address the problem, the residual attention mechanism of the proposed algorithm is in Equation (3),

(3) $$A_{i,c}\left(x\right)=\left(1+M_{i,c}\left(x\right)\right)T_{i,c}\left(x\right)$$

where *M*(*x*) is the output of soft attention branch and *T*(*x*) is the output of main branch. When *M*(*x*) = 0, the input of this layer is equal to *T*(*x*). Therefore, the effect of this layer cannot be worse than the original *T*(*x*). By adding one to *M*(*x*), it makes the feature maps from main branch more prominent and more discriminative and makes the network to easily reach a very deep level and have a good performance.

### 2.4. Mixed Loss Function

The Dice loss function used in the proposed algorithm is inspired by V-Net [13], as shown in Equation (4),

(4) $$L_{dice}=1-2\times \frac{\sum_{i=1}^{N}p_{i}g_{i}+\epsilon }{\sum_{i=1}^{N}p_{i}+\sum_{i=1}^{N}g_{i}+\epsilon }$$

where *N* indicates the number of all predicted voxels. $p_{i}$ represents the probability that the predicted voxel *i* belongs to class *P*, $g_{i}$ represents the voxel *i* in the feature map. ϵ is $10^{-4}$ in the proposed algorithm. The gradient relationship is in Equation (5).

(5) $$\frac{\partial L_{d}\left(P,G\right)}{\partial p_{k}}=-2\times \frac{\sum_{i=1}^{N}p_{i}g_{i}-g_{k}\sum_{i=1}^{N}\left(p_{i}+g_{i}\right)}{{\left[\sum_{i=1}^{N}\left(p_{i}+g_{i}\right)\right]}^{2}}$$

The cross-entropy loss function is in Equation (6).

(6) $$L_{c}=-\frac{1}{N}\overset{N}{\underset{i=1}{\sum }}\overset{3}{\underset{c=1}{\sum }}w_{i}^{c}y_{i}^{c}\log{\hat{y_{i}}}^{c}$$

Therefore, the final loss function of the proposed algorithm is in Equation (7).

(7) $$L_{total}=\lambda \times L_{dice}+L_{c}$$

where *λ* = 0.5.

## 3. Experimental Results

### 3.1. Experimental Environment and Parameters

The experimental hardware and software configuration in this paper is shown in Table 1. The training hyperparameter settings in the two stages of the proposed algorithm are shown in Table 2.

### 3.2. Data Sets and Quantitative Evaluation Metrics

All CT images used in this experiment are from the Liver Tumor Segmentation Challenge of the 2017 International Conference on Medical Image Computing and Computer-Assisted Intervention (MICCAI). This data set consists of subjects from six hospitals with different types of liver tumor diseases, including 131 sets of enhanced CT image sequences. Each CT sequence covers the entire abdomen, using the Nifti format; the number of axial slices is not fixed, ranging from 74 to 987. The resolution of each CT slice is 512 × 512, the pixel interval is from 0.56 mm to 1.0 mm, and the slice interval is from 0.45 mm to 6.0 mm. The data set also provides the ground-truth segmentation results of liver and liver tumors manually annotated by clinicians.

In the experiment, we divided the 131 abdominal CT image sequences into a training set (81 sequences), validation set (25 sequences), and test set (25 sequences), and we used random translation, random rotation, and arbitrary scale transformation as the data augmentation methods.

The Dice coefficient is a standard evaluation metric in medical image segmentation; therefore, we used Dice as the quantitative metric in the experiment. The calculation formula of the Dice coefficient is in Equation (8).

(8) $$\mathrm{Dice}\left(P,G\right)=\frac{2\left|\;P\;\cap \;G\right|}{\left|P\right|\;+\;\left|G\right|}$$

where *P* and *G* represent the proposed algorithm’s segmentation results and the ground-truth segmentation results, respectively. The range of the Dice coefficient is between 0 and 1, and the larger the Dice coefficient is, the higher the segmentation accuracy is.

### 3.3. Training and Verification of the Network Model 

**Figure 7:**
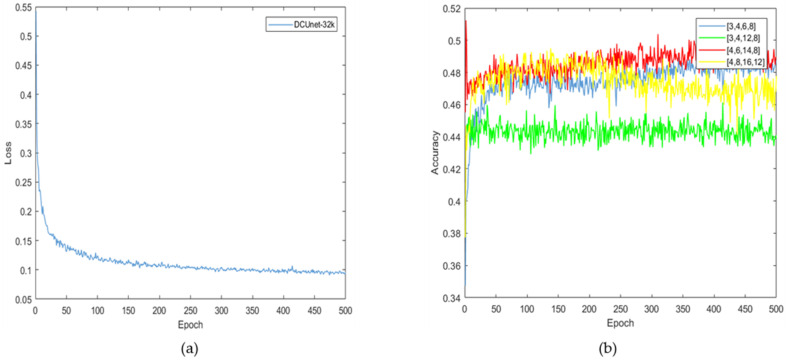
The learning process and the experimental results of the model: (**a**) the loss function of the proposed algorithm; (**b**) the results of different micro blocks.

The loss function curve of the proposed algorithm is shown in Figure 7a. It can be seen that the loss value is reduced to 0.1 after 500 rounds of training iteration, which indicates that the trained deep learning network model has converged.

In the liver localization stage, the number of micro blocks in dense blocks is an important parameter for the performance of the neural network. To optimize the effectiveness of this parameter, we compare five selections, which are (4, 6, 14, 8), (3, 4, 6, 8), (3, 4, 12, 8), (4, 6, 14, 8), and (4, 8, 16, 12), respectively, and the results are shown in Figure 7b. When the micro block numbers are (4, 8, 16, 12), the model’s accuracy first stabilized and then gradually declined, which indicates that the micro block numbers with larger values may cause over-fitting and reduce the neural network’s performance. When the micro block numbers are (3, 4, 6, 8), the curve of the segmentation accuracy is the lowest, which indicates that the micro block numbers with smaller values cannot obtain sufficient parameters to accurately segment liver and tumors. Therefore, based on the experimental results, the proposed algorithm set the micro block numbers as (4, 6, 14, 8).

### 3.4. The Results and Analysis of This Algorithm

**Figure 8:**
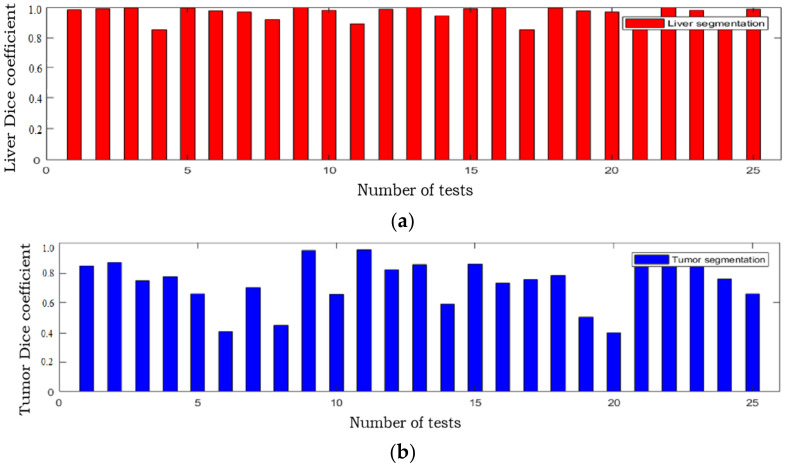
The Dice coefficient of the segmentation results of the proposed algorithm in the test set on 25 CT sequence images: (**a**) Dice coefficient of the liver segmentation results; (**b**) Dice coefficient of the tumor segmentation results.

The Dice coefficient of the two stages of the proposed algorithm on the test set is shown in Table 3. The Dice coefficients of liver and tumor on the training and testing data set is shown in Table 4. There are 25 samples in the test data set, and the Dice coefficient values of the liver and liver tumor segmentation results from all these 25 samples are shown in Figure 8. The Dice coefficient of the liver segmentation result is mostly around 0.95, and the Dice coefficient of the tumor segmentation result is mostly around 0.8, indicating that the proposed algorithm can accurately segment liver and liver tumors.

**Figure 9:**
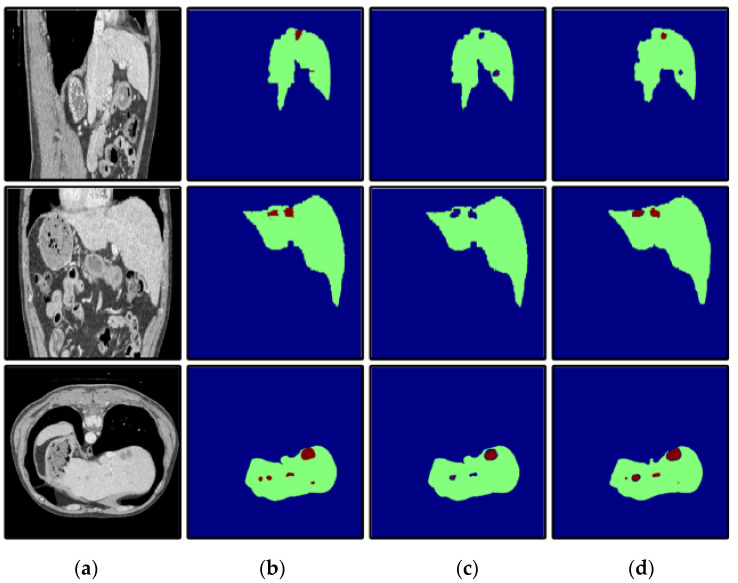
The influence of the attention mechanism on liver tumor segmentation results; the first row is the sagittal plane, the second row is the coronal plane, and the third row is the transverse plane. (**a**) CT original images; (**b**) the real segmentation results; (**c**) DCUNet-noAttention segmentation results; (**d**) DCUNet-Tumor segmentation results.

To verify the effectiveness of the attention mechanism, we compared the segmentation results of DCUNet-noAttention (without attention mechanism) and DCUNet-Tumor (with attention mechanism). The segmentation results of the two models are in Figure 9, and DCUNet-Tumor can obtain a more accurate segmentation result of liver and liver tumors, and its segmented liver has no extra holes. However, DCUNet-noAttention cannot detect small tumor regions and only identify the approximate location of the tumor region, resulting in poor performance.

**Figure 10:**
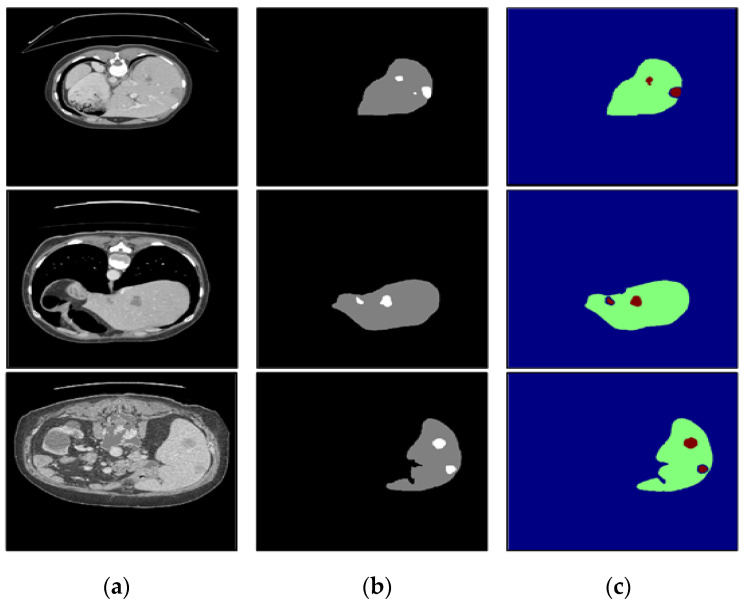
The segmentation results of our algorithm for small liver tumors: (**a**) CT original images; (**b**) the real segmentation results; (**c**) our segmentation results.

**Figure 11:**
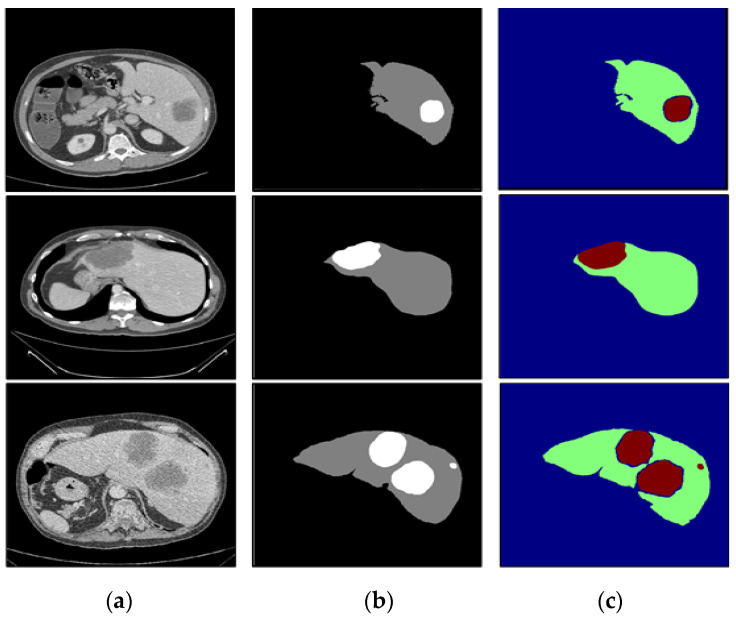
The segmentation results of our algorithm for large liver tumors: (**a**) CT original images; (**b**) the real segmentation results; (**c**) our segmentation results.

**Figure 12:**
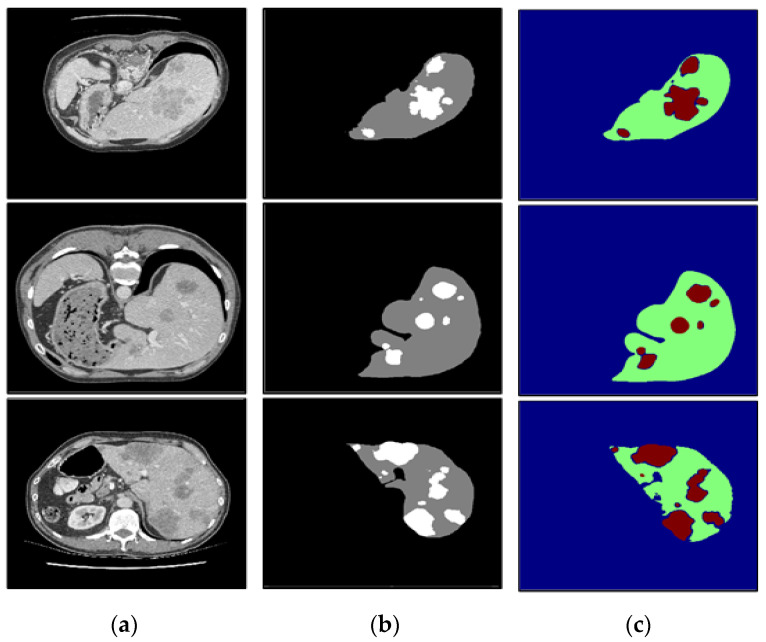
The segmentation results of our algorithm for multiple liver tumors: (**a**) CT original images; (**b**) the real segmentation results; (**c**) our segmentation results.

To verify the performance under different circumstances, we test the proposed algorithm on the liver CT images with small tumors, large tumors, and multiple tumors, and the experimental results are shown in Figure 10, Figure 11 and Figure 12, respectively.

In this paper, we paid more attention to small tumor detection. In the LiTS data set, there are 46 liver CT volumes with small tumors; the accurate detection percentage of the proposed algorithm was 38/46, or 82.6%, and its Dice coefficient is 0.68. Besides, Figure 10 shows the segmentation results of the small tumor regions, and we find that the proposed algorithm can detect the small tumors accurately without any miss-segmentation problems, and there are no extra holes in the segmented liver. Figure 11 shows the segmentation results of the large tumor regions, and we find that the segmentation results for the liver and liver tumors are accurate. Figure 12 shows the segmentation results of multiple tumors, and we find that the tumor located in different positions of the liver can be detected. All the experimental results indicate the ability of the proposed algorithm to detect and segment various types of liver tumors.

**Figure 13:**
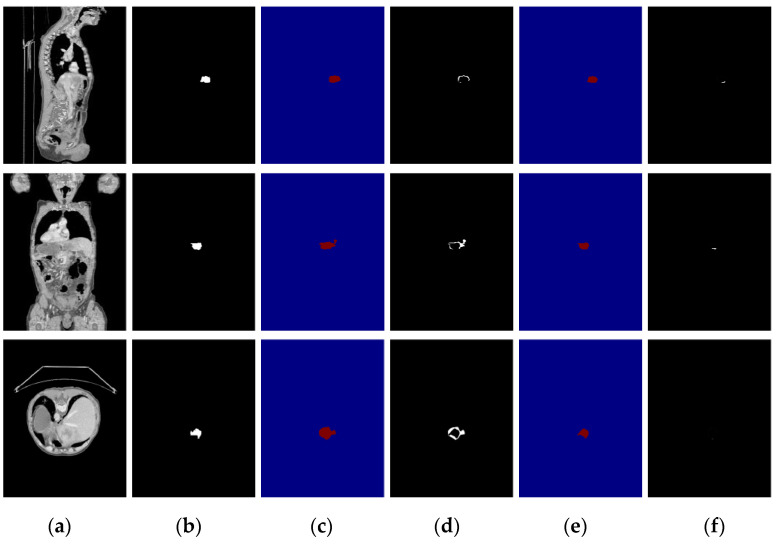
Segmentation result of a small tumor; the first row is the sagittal plane, the second row is the coronal plane, and the third row is the transverse plane. (**a**) CT image; (**b**) Ground truth; (**c**) DenseUNet segmentation results; (**d**) difference image between DenseUnet and ground truth; (**e**) the proposed algorithm’s segmentation results; (**f**) difference image between the proposed algorithm and ground truth.

**Figure 14:**
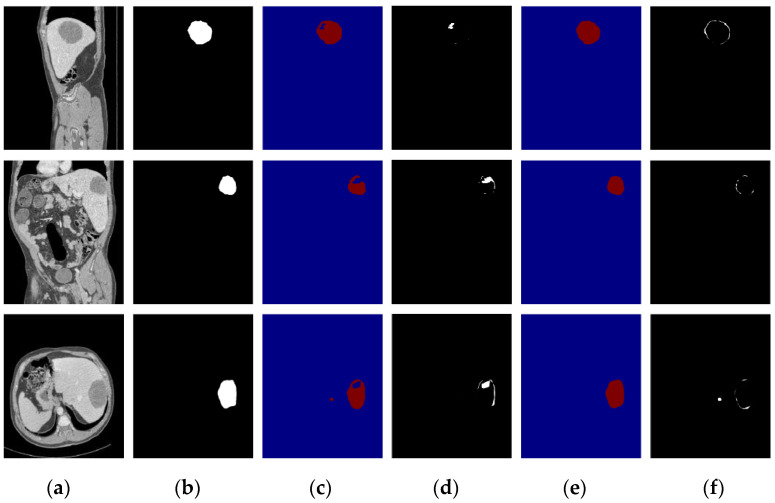
Segmentation result of a large tumor; the first row is the sagittal plane, the second row is the coronal plane, and the third row is the transverse plane. (**a**) CT image; (**b**) Ground truth; (**c**) DenseUNet segmentation results; (**d**) difference image between DenseUnet and ground truth; (**e**) the proposed algorithm’s segmentation results; (**f**) difference image between the proposed algorithm and ground truth.

**Figure 15:**
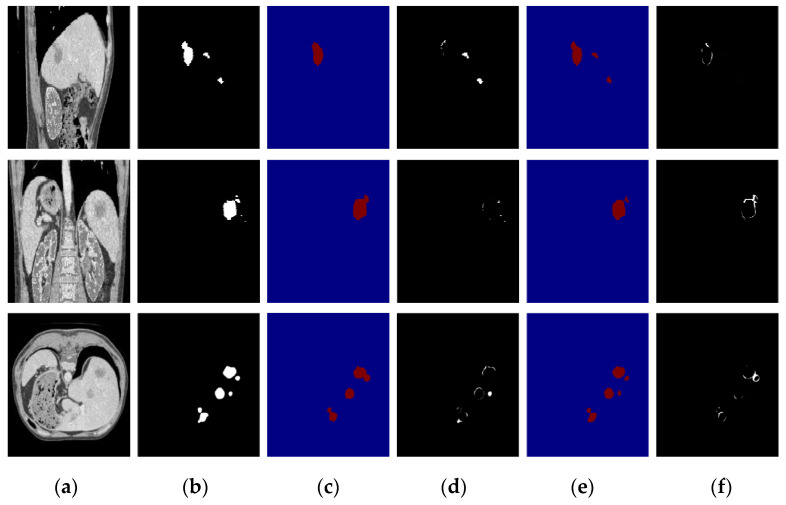
Segmentation result of a multiple-tumor area; the first row is the sagittal plane, the second row is the coronal plane, and the third row is the transverse plane. (**a**) CT image; (**b**) Ground truth; (**c**) DenseUNet segmentation results; (**d**) difference image between DenseUnet and ground truth; (**e**) the proposed algorithm’s segmentation results; (**f**) difference image between the proposed algorithm and ground truth.

Furthermore, we compare the segmentation results of the proposed algorithm with those of DenseUNet [14], and the results of large tumors, small tumors, and multiple tumors are shown in Figure 13, Figure 14 and Figure 15, respectively. Based on the comparison results, we find that DenseUNet segmentation results have some problems, such as many under-segmented regions in the outer contour of the tumor and holes in the center of the tumor, so there are big differences between the ground truth and DenseUNet. In contrast, the proposed algorithm can accurately detect and segment the liver tumor regions, and the differences between the ground truth and the proposed algorithm’s segmentation results are very small.

In this paper, we propose a two-stage liver and liver tumor segmentation algorithm for abdominal CT images, and the two stages are DCUNet-Liver and DCUNet-Tumor. We added the attention mechanism to improve the accuracy of segmenting small tumors. Experimental results show that the algorithm in this paper can accurately segment liver and liver tumors, and the Dice coefficients reached 0.967 and 0.725, respectively. Compared with other state-of-the-art algorithms, the proposed algorithm has a better segmentation effect, faster calculation speed, and requires fewer computational resources.

## 4. Discussion

We trained the proposed algorithm using the experimental environment shown in Table 1, which takes 35 h, and we compared the proposed algorithm with DenseUNet [14] in terms of the parameter amount and calculation speed, shown in Table 5. From the comparison results, we conclude that the proposed algorithm reduces the parameters of the neural network model and improves the computational speed.

We quantitatively compared the proposed algorithm with other state-of-the-art liver and tumor segmentation algorithms. As shown in Table 6, the proposed algorithm has an excellent liver and tumor segmentation performance and outperforms the others.

Moreover, we compared the proposed algorithm with the other methods submitted by MICCAI 2017, including 13 groups of liver segmentation results and tumor segmentation results (https://competitions.codalab.org/competitions/17094#results (accessed on 15 December 2020)). All methods used the same dataset, named LiTS. The comparison results are shown in Table 7 and Table 8. From these two tables, we conclude that the Dice value of our algorithm for liver and liver tumors is the highest, reaching 0.967 and 0.725. In addition, the VOE and RVD coefficient of the liver was 0.082 and 0.022, and for liver tumors was 0.347 and 0.034.

We asked radiologists to manually make the liver tumor segmentation, and obtained a Dice coefficient for the human raters of about 0.78, while that of the proposed algorithm is 0.725. Although the accuracy of our algorithm is slightly lower than that of manual detection, the use of an automatic segmentation algorithm can greatly liberate manpower and reduce the pressure on doctors.

## Figures and Tables

**Table 1:** Experimental hardware and software configuration.

Environment	Configuration Information
GPU	Tesla K40L
Video memory	12 G
Memory	64 G
Operating system	Ubuntu 16.04
Hard disk	4 TB
Programming Software	Keras 2.2.0; Python 2.7; Matlab 2015b

**Table 2:** Training hyperparameters of each module in the two stages of the algorithm.

Hyperparameters	Setting	
	Liver Localization Module	Tumor Segmentation Module
Initial learning rate	0.001	0.001
Dropout	0.5	0.5
Batch_size	10	1
Epoch	500	500
Optimizer	SGD	SGD
Growth_rate	32	64

**Table 3:** The Dice coefficient of the liver and tumor at various stages.

Network Model	Liver (Dice)	Tumor (Dice)
DCUnet-Liver	0.934	0.656
DCUnet-Tumor	0.967	0.725

**Table 4:** The Dice coefficient of liver and tumor on the training and the testing dataset.

Network Model	Training (Dice)	Testing (Dice)
DCUnet-Liver	0.99	0.967
DCUnet-Tumor	0.86	0.725

**Table 5:** Comparison of the parameter quantity and calculation efficiency of the network model.

Methods	Number of Parameters	Time (Seconds/Piece)
2D DenseUNet [14]	49,970,531	0.674
H-DenseUNet [14]	61,444,622	0.829
Our algorithm	21,909,838	0.479

**Table 6:** Comparison of the Dice coefficients between our algorithm and other algorithms.

Network Model	Liver	Tumor
Li [14]	0.961	0.722
Bi [15]	0.934	0.645
Yuan [16]	0.963	0.657
Kaluva [17]	0.912	0.492
Vorontsov [18]	0.951	0.661
Liu [19]	0.951	—
Guo [20]	0.943	—
Meng [21]	0.965	0.689
Fang [22]	0.961	—
Our algorithm	**0.967**	**0.725**

Bold indicates the highest values.

**Table 7:** Comparison of liver segmentation.

Group	Dice	VOE	RVD
**Ours**	**0.967**	**0.082**	**0.022**
Mantis_shrimp	0.959	0.078	0.009
schwein	0.959	0.078	0.008
SMC_QMIA	0.958	0.079	−0.023
Yong	0.958	0.081	0.030
BriceRauby	0.957	0.083	0.015
Karo	0.955	0.085	0.034
kikikirai	0.955	0.086	−0.029
Neymo	0.954	0.086	−0.009
VincentHan	0.953	0.088	−0.001
CYNSAHZU	0.950	0.084	−0.006
kirai	0.946	0.1	−0.022
Jangho_Kwon	0.937	0.109	−0.021
EdwardMa	0.924	0.141	−0.025

Bold indicates the highest values.

**Table 8:** Comparison of liver tumor segmentation.

Group	Dice	VOE	RVD
**Ours**	**0.725**	**0.347**	**−0.034**
SMC_QMIA	0.707	0.333	−0.096
davidlinhl	0.7	0.342	−0.064
CYNSAHZU	0.699	0.367	−0.136
KristinChen	0.694	0.401	−0.195
MengLei1	0.69	0.362	−0.069
Cerry	0.69	0.370	−0.052
viggin	0.689	0.400	−0.162
LeoZ	0.686	0.376	0.014
Eric101	0.681	0.353	−0.066
Yong	0.661	0.375	−0.007
zhoushen	0.645	0.366	−0.082
hyukist	0.631	0.375	−0.088
mahendrakhened	0.556	0.435	7.179

Bold indicates the highest values.
